# Extralaryngeal branching of the recurrent laryngeal nerve: a meta-analysis of 28,387 nerves

**DOI:** 10.1007/s00423-016-1455-7

**Published:** 2016-06-02

**Authors:** Brandon Michael Henry, Jens Vikse, Matthew J. Graves, Silvia Sanna, Beatrice Sanna, Iwona M. Tomaszewska, R. Shane Tubbs, Krzysztof A. Tomaszewski

**Affiliations:** 1 International Evidence-Based Anatomy Working Group, 12 Kopernika St, 31-034 Krakow, Poland; 2 Department of Anatomy, Jagiellonian University Medical College, 12 Kopernika St, 31-034 Krakow, Poland; 3 Department of Surgical Sciences, University of Cagliari, S.S. 554, Bivio Sestu, 09042 Monserrato, CA, Sardinia Italy; 4 Faculty of Medicine and Surgery, University of Cagliari, S.S. 554, Bivio Sestu, 09042 Monserrato, CA, Sardinia Italy; 5 Department of Medical Education, Jagiellonian University Medical College, 16 św. Łazarza Street, 31-530 Krakow, Poland; 6 Seattle Science Foundation, 550 17th Ave, James Tower, Suite 600, Seattle, WA 28122 USA

**Keywords:** Recurrent laryngeal nerve, Extralaryngeal branching, Thyroid, Surgery, Anatomic variations

## Abstract

**Introduction:**

The recurrent laryngeal nerves (RLN) are branches of the vagus nerve that go on to innervate most of the intrinsic muscles of the larynx. Historically, the RLN has been considered to branch after it enters the larynx, but numerous studies have demonstrated that it often branches before. The wide variability of this extralaryngeal branching (ELB) has significant implications for the risk of iatrogenic injury. We aimed to assess the anatomical characteristics of ELB comprehensively.

**Methods:**

Articles on the ELB of the RLN were identified by a comprehensive database search. Relevant data were extracted and pooled into a meta-analysis of the prevalence of branching, branching pattern, distance of ELB point from the larynx, and presence of positive motor signals in anterior and posterior ELB branches.

**Results:**

A total of 69 articles (*n* = 28,387 nerves) from both intraoperative and cadaveric modalities were included in the meta-analysis. The overall pooled prevalence of ELB was 60.0 % (95 % CI 52.0–67.7). Cadaveric and intraoperative subgroups differed with prevalence rates of 73.3 % (95 % CI 61.0–84.0) and 39.2 % (95 % CI 29.0–49.9), respectively. Cadavers most often presented with a ELB pattern of bifurcation, with a prevalence of 61.1 %, followed by no branching at 23.4 %. Branching of the RLN occurred most often at a distance of 1–2 cm (74.8 % of cases) prior to entering the larynx. A positive motor signal was most often noted in anterior RLN branches (99.9 %) but only in 1.5 % of posterior branches.

**Conclusions:**

The anatomy of the RLN is highly variable, and ELB is likely to have been underreported in intraoperative studies. Because of its high likelihood, the possibility of ELB needs to be assessed in patients to prevent iatrogenic injury and long-term postoperative complications.

**Electronic supplementary material:**

The online version of this article (doi:10.1007/s00423-016-1455-7) contains supplementary material, which is available to authorized users.

## Introduction

The recurrent laryngeal nerves (RLN) are branches of the vagus nerve, which classically arise in the inferior neck and innervate the intrinsic muscles of the larynx except for the cricothyroid muscle [1]. However, it has recently been shown that the RLN can also contribute significantly to the innervation of the cricothyroid muscle [2]. Its terminal branches must split in order to innervate their respective muscles, but this branching can occur anywhere from several centimeters from the inferior rim of the cricothyroid joint to within the larynx itself [3–8]. The terminal branch of the RLN as it courses superior to the cricothyroid joint is commonly deemed the inferior laryngeal nerve. It is of paramount importance that the RLN and its extralaryngeal branches (ELB), if present, are carefully dissected and identified during procedures in the anterior neck. Failure to identify these neural structures, or inadequate knowledge of their variability, can lead to an increased incidence of iatrogenic nerve injury [9]. As described by Kandil et al. in 2011 [10], the RLN typically branches superior to the inferior thyroid artery and posterolaterally to the ligament of Berry, and this is the location where the nerve is most susceptible to injury. If the posterior branch is identified and believed to be the sole RLN, the anterior branch is particularly vulnerable to injury when the capsular dissection approach to thyroidectomy is used [4]. If the anterior branch is identified first, it is more likely that the surgeon will find the posterior branch during capsular dissection, thereby preventing injury [4]. The likelihood of lesion to the anterior branch is particularly important because there is a high risk of vocal cord palsy and long-term complications from its injury.

Data on the prevalence of ELB have been debated for years, with reported prevalence rates ranging widely from around 5 % [11] to 100 % [12–21]. The rates also differ depending on whether the studies were conducted intraoperatively or on cadavers. Since the RLN is very susceptible to injury in a multitude of procedures, a thorough and complete understanding of its variability and the associated implications is crucial for preventing iatrogenic injuries and long-term complications. The aim of our analysis was to provide a comprehensive and evidence-based assessment of the ELB of the RLN. An accurate and complete assessment of the ELB is necessary to provide a complete understanding of the risk factors associated with neck surgery and the vital importance of taking precautionary measures to prevent injury-related complications.

## Methods

### Search strategy

To identify articles for inclusion in the meta-analysis, searches were performed through December 2015 in the following databases: PubMed, EMBASE, ScienceDirect, China National Knowledge Infrastructure (CNKI), SciELO, BIOSIS, and Web of Science. The comprehensive search strategy applied to PubMed is presented in Table 1. No date or language restrictions were imposed. In order to identify additional studies eligible for the meta-analysis, the references of all included articles were thoroughly searched. Throughout the meta-analysis, the Preferred Reporting Items for Systematic Reviews and Meta-Analyses (PRISMA) guidelines were strictly followed (Online Resource 1) [22]. Our study was prospectively registered in the PROSPERO database (CRD42015026096).

**Table 1:** Search terms and strategy for PubMeb

1	(((“recurrent laryngeal nerve”[Title/Abstract]) OR “nervus laryngeus recurrens”[Title/Abstract]) OR “inferior laryngeal nerve”[Title/Abstract]) OR “inferior thyroid artery”[Title/Abstract]
2	((((((“anatomy”[Title/Abstract]) OR “variation”[Title/Abstract]) OR “anomaly”[Title/Abstract]) OR “course”[Title/Abstract]) OR “relationship”[Title/Abstract]) OR “branching”[Title/Abstract]) OR “division”[Title/Abstract]
3	1 AND 2
4	(“recurrent laryngeal nerve/anatomy and histology”[MeSH Major Topic])
5	“non recurrent laryngeal nerve”[Title/Abstract]
6	“Zuckerkandl’s Tubercle”
7	“Galen’s anastomosis” OR “Arytenoid plexus” OR “Cricoid anastomosis” OR “Thyroarytenoid anastomosis” OR “cricothyroid anastomosis” OR “human communicating nerve”
8	3 OR 4 OR 5 OR 6 OR 7

### Eligibility assessment

Eligibility of studies for inclusion in the meta-analysis was assessed by three independent reviewers (JV, MJG, and SS). All cadaveric or intraoperative studies that reported extractable prevalence data with respect to rate of ELB were included. The exclusion criteria included case reports, case series, letters to the editor, or conference abstracts. Studies on human fetuses or involving patients with congenital anomalies of or trauma to the head and neck region were also excluded. All studies published in languages not fluently spoken by any of the authors were translated by medical professionals fluent in both English and the language of the manuscript. Any disagreements between reviewers arising during the eligibility assessment process were resolved by consensus.

### Data extraction

Data from the included studies were independently extracted by three reviewers (BMH, JV, and SS). The extracted data included year, country, sample size (number of nerves), prevalence of ELB, symmetry of ELB, type of ELB (no branching, bifurcation, trifurcation, multiple branches), the distance from the ELB site to the inferior rim of the cricothyroid joint (0–1, 1–2, 2–3, 3–4 cm), and the intraoperative electrophysiologically assessed prevalence of positive motor signals in the anterior and posterior branches of ELB RLNs. In the event of any discrepancies in the data, the authors of the original were contacted for clarification when possible.

### Statistical analysis

The single-categorical and multi-categorical pooled prevalence rates of the ELB of the RLN were calculated by BMH and JV using MetaXL version 2.0 by EpiGear Pty Ltd. (Wilston, Queensland, Australia) [23]. A random effects model was used for all statistical analyses. Heterogeneity was assessed by both the chi^2^ test and the *I* ^2^ statistic. For the chi^2^ test, a *p* value of <0.10 for Cochran’s *Q* served as an indicator of significant heterogeneity among the studies analyzed [24]. The results of the *I* ^2^ statistic were interpreted as follows: 0–40 % might not be important; 30–60 % could indicate moderate heterogeneity; 50–90 % could indicate substantial heterogeneity; and 75–100 % could represent considerable heterogeneity [24].

Subgroup analysis was performed on the basis of type of study (cadaveric vs. intraoperative), study design (prospective vs. retrospective), geographical origin of the study, gender, and side (left vs. right). Data on subgroups was maximally extracted based on its availability within the analyzed studies. Significant differences between analyzed groups were determined by their confidence intervals. If the confidence intervals of any two rates overlapped, the differences were regarded as statistically insignificant [23]. Lastly, sensitivity was assessed by a leave-one-out analysis to probe further for potential sources of heterogeneity.

## Results

### Study identification

**Fig. 1:**
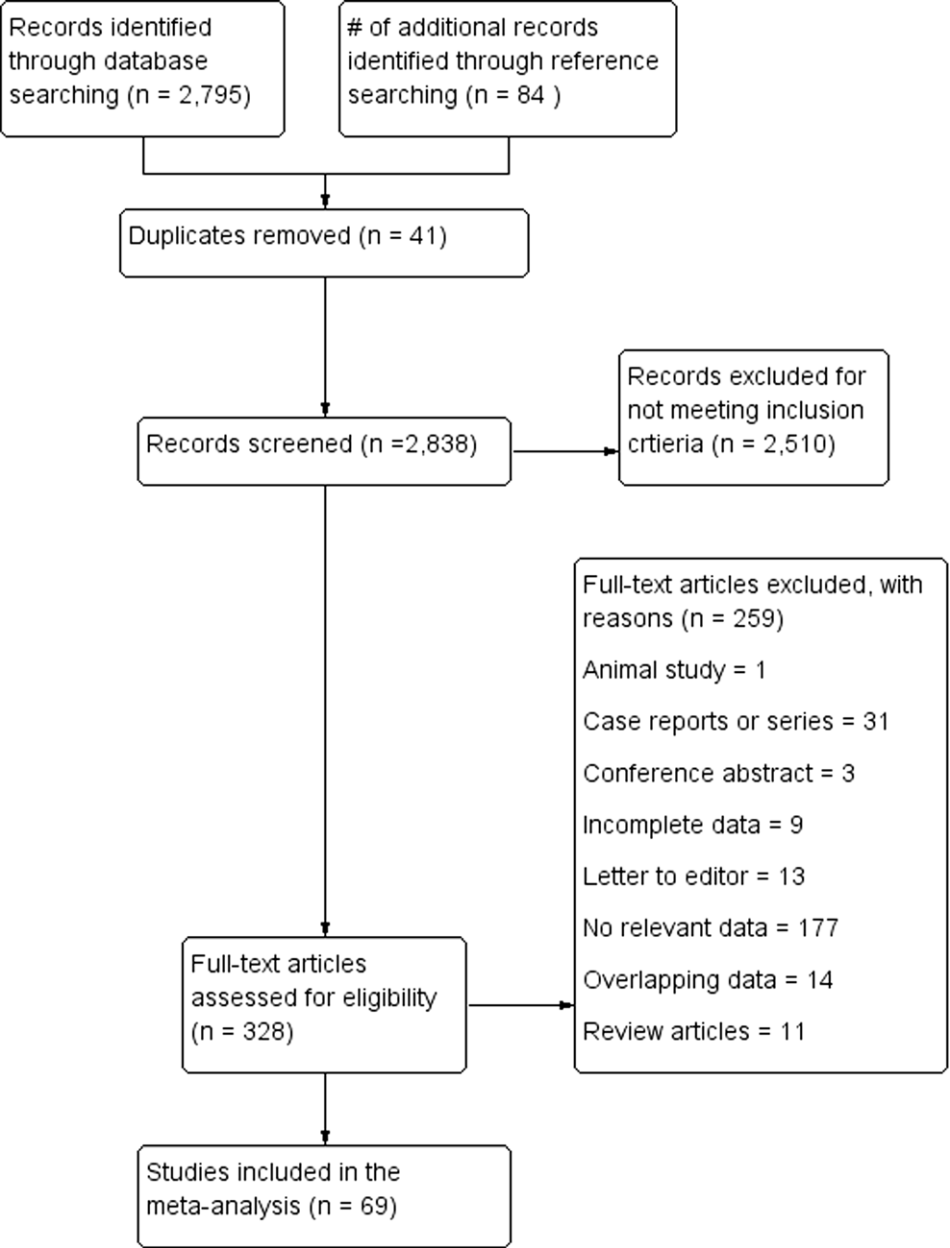
PRISMA flow chart of study identification and inclusion in the meta-analysis

The flow of studies through the meta-analysis is presented in Fig. 1. The search of the major electronic databases identified an initial 2795 articles, with a further 84 identified in the search through the references of those studies. A total of 328 articles were assessed for eligibility using full texts, of which 259 were excluded and 69 were included in the meta-analysis.

### Characteristics of included studies

The characteristics of the studies included in the meta-analysis are summarized in Table 2. A total of 69 studies [3–7, 9–21, 25–75] were included (*n* = 28,387 total nerves): 26 intraoperative, 42 cadaveric, and 1 that included both intraoperative and cadaveric subjects [50]. Among the intraoperative studies, 16 were prospective and 10 were retrospective. The dates of the included studies ranged from 1921 [13] to the end of year 2015 [30, 41, 66]. The studies demonstrated a wide range of geographical origin, with the most substantial contributions coming from Asia (22 studies), Europe (25), and North America (13). Fourteen studies in Chinese, one in French, one in Italian, and one in Portuguese were translated into English and included in our analysis.

**Table 2:** Table of included studies

Study	Country	Type	*n* (no. of nerves)	% of ELB
Al-Salihi and Dabbagh [25]	Iraq	C	212	25.5
Altorjay et al. [26]	Hungary	IP	1023	51.5
Ardito et al. [27]	Italy	IP	2615	72.4
Armstrong and Hinton [28]	USA	C	100	73.0
Asgharpour et al. [3]	Spain	C	284	54.6
Barczyński et al. [29]	Poland	IP	302	22.2
Barczyński et al. [30]	Poland	IP	2500	24.5
Bargy et al. [31]	France	C	56	10.7
Beneragama and Serpell [4]	Australia	IP	213	40.4
Bowden [32]	Great Britain	C	54	77.8
Cakir et al. [33]	Turkey	C	130	58.5
Cernea et al. [34]	Brazil	IR	2154	64.5
Chang [12]	China	C	50	100
Chen et al. [35]	China	C	90	68.9
Chen et al. [36]	China	C	94	69.1
Clader et al. [37]	USA	C	50	58.0
Dai et al. [38]	China	IR	339	59.9
Dilworth [13]	England	C	66	100
Fontenot et al. [39]	USA	IR	719	36.7
Gurleyik [40]	Turkey	IP	200	27.0
Gurleyik [41]	Turkey	IP	185	33.0
Hisham and Lukman [42]	Malaysia	IP	490	34.1
Hsu et al. [14]	China	C	177	100
Iqbal and Zumair [43]	Pakistan	IR	93	58.1
Jiang et al. [44]	China	IR	292	63.4
Kandil et al. [10]	USA	IP	310	42.9
Katz and Nemiroff [45]	USA	IP	1177	63.5
Keros and Nemanić [15]	Croatia	C	300	100
King and Gregg [46]	USA	C	43	27.9
Kulekci et al. [47]	Turkey	C	194	80.4
Kuo et al. [48]	China	C	100	62.0
Laux and Guerrier [49]	France	C	200	43.0
Lu et al. [50]	China	C + IR	66	27.3
Makay et al. [9]	Turkey	IP	253	24.1
Matubis et al. [51]	Philippines	C	108	14.8
Moreau et al. [52]	France	C	34	29.4
Morrison [53]	USA	C	200	43.0
Nemiroff and Katz [5]	USA	IP	153	41.2
Ngo Nyeki et al. [54]	Cameroon and Gabon	IP	62	9.7
Nguyen et al. [55]	France	C	60	86.7
Norland [56]	USA	C	62	96.8
Page et al. [57]	France	IP	403	19.4
Pascoal et al. [58]	Brazil	C	44	70.5
Pichler and Gisel [16]	Austria	C	100	100
Pradeep et al. [59]	India	IR	583	30.5
Prior and Fasce [60]	Italy	C	100	11.0
Reed [11]	USA	C	506	5.3
de Souza 1981 [61]	Brazil	C	98	25.5
Rueger [17]	USA	C	19	100
Rustad [62]	USA	C	200	43.0
Salama and McGrath [6]	Australia	C	144	65.3
Schweizer and Dörfl [7]	Switzerland	C	42	88.1
Serpell et al. [63]	Australia	IP	838	25.7
Serpell [64]	Australia	IR	977	24.7
Shao et al. [65]	China	IP	4241	8.6
Shao et al. [66]	China	IR	2869	11.2
She et al. [67]	China	C	200	42.0
She et al. [18]	China	C	100	100
Sun et al. [68]	China	C	100	94.0
Sunderland and Swaney [69]	Australia	C	130	70.0
Tang et al. [70]	China	C	160	91.9
Wang et al. [71]	China	IR	63	76.2
Weeks and Hinton [72]	USA	IR	17	88.2
Williams [19]	England	C	100	100
Yalcin et al. [73]	Turkey	C	96	92.7
Yalcin et al. [74]	Turkey	C	120	93.3
Yang et al. [20]	China	C	90	100
Yuan [75]	China	C	117	67.5
Zhou et al. [21]	China	C	120	100

*ELB* extralaryngeal branching, *C* cadaveric, *IP* intraoperative prospective, *IR* intraoperative retrospective

### Prevalence of extralaryngeal branching

**Fig. 2:**
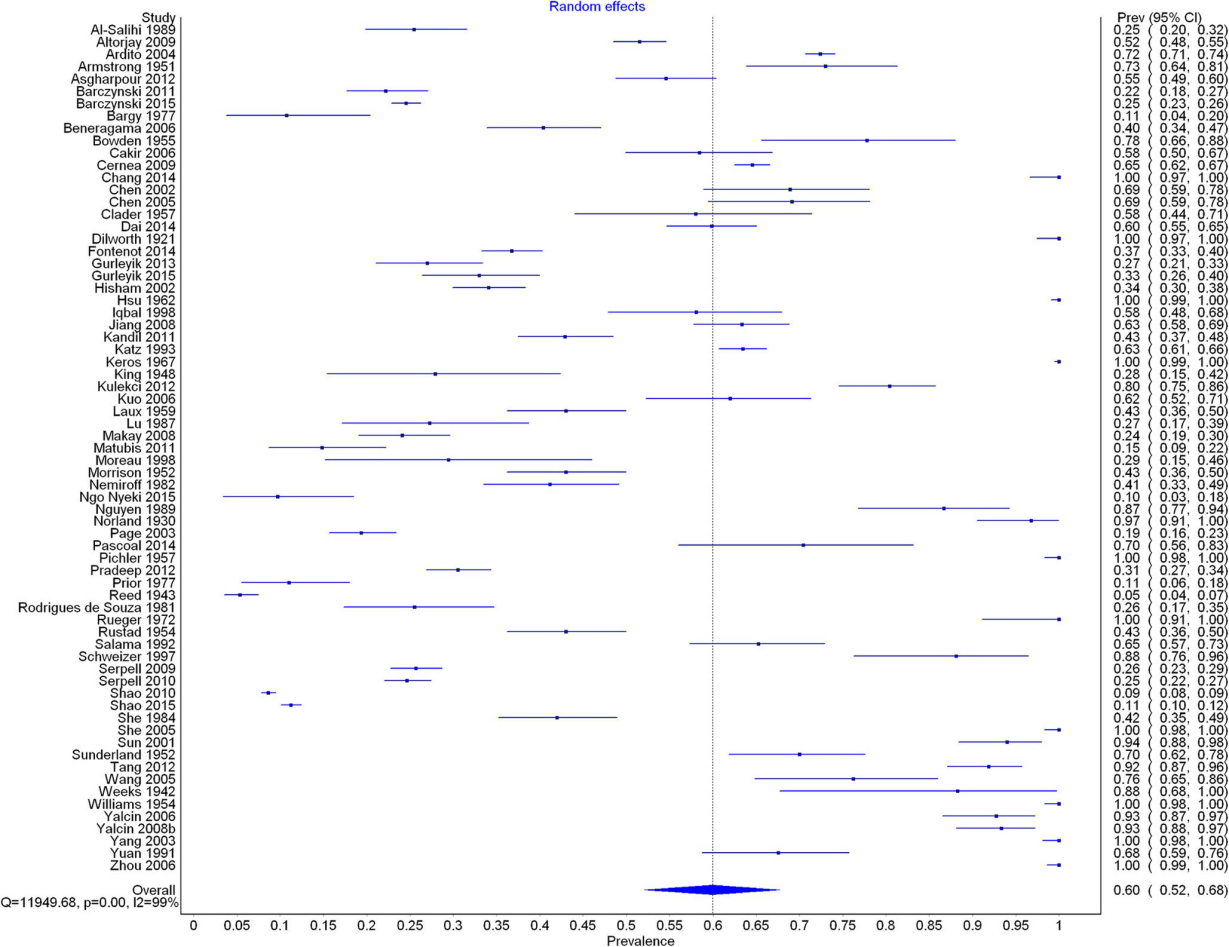
Forest plot for prevalence of extralaryngeal branching of the recurrent laryngeal nerve

A total of 69 studies (*n* = 28,387 nerves) reported data on ELB prevalence. The overall pooled prevalence rate of ELB was 60.0 % (95 % CI 52.0–67.7) (Fig. 2). Subgroup analysis by type of study revealed significant differences in the prevalence of ELB between cadaveric (73.3 %; 95 % CI 61.0–84.0) and intraoperative (39.2 %; 95 % CI 29.0–49.9) studies. No significant differences were found between subgroups with respect to side (left vs. right), gender, or geographical origin. Details of subgroup analyses are presented in Table 3. No significant differences were observed in the sensitivity analysis.

**Table 3:** Subgroup analysis for the prevalence of extralaryngeal branching

Subgroup	No. of studies (no. of nerves)	Pooled prevalence of ELB % (95 % CI)	*I* ^2^: % (95 % CI)*
Overall	69 (28,387)	60.0 (52.0–67.7)	99.4 (99.4–99.5)
Cadaveric	42 (5250)	73.3 (61.0–84.0)	98.9 (98.8–99.0)
Intraoperative	26 (23,071)	39.2 (29.0–49.9)	99.6 (99.6–99.6)
Intraoperative (prospective)	16 (14,965)	33.4 (20.5–47.7)	99.7 (99.6–99.7)
Intraoperative (retrospective)	10 (8106)	50.2 (32.0–68.4)	99.6 (99.5–99.6)
Left sides	29 (6443)	56.6 (43.6–69.2)	98.9 (98.8–99.1)
Right sides	30 (6561)	58.5 (45.1–71.3)	99.0 (98.9–99.1)
Males	6 (420)	59.6 (20.0–87.8)	97.3 (95.8–98.2)
Females	6 (794)	59.7 (22.7–92.0)	98.1 (97.1–98.7)
Asia	23 (10,754)	66.1 (50.2–80.4)	99.5 (99.5–99.6)
Europe	24 (9417)	62.7 (49.2–75.3)	99.3 (99.2–99.4)
North America	12 (3456)	55.8 (39.3–71.7)	98.7 (98.4–99.0)
Oceania	5 (2302)	44.3 (29.0–60.2)	97.9 (96.8–98.7)
South America	3 (2296)	53.4 (25.3–80.5)	96.7 (93.3–98.4)

*p value for Cochran’s Q for all subgroups was <0.001

A total of six studies (*n* = 641 subjects) reported data on the symmetry of the RLN with respect to ELB. The RLN was found to be symmetrical in 36.5 % (95 % CI 16.1–59.6) of individuals (*I* ^2^ = 96.6 % (95 % CI 94.5–97.9); *p* < 0.001).

### Prevalence of the types of extralaryngeal branching

**Fig. 3:**
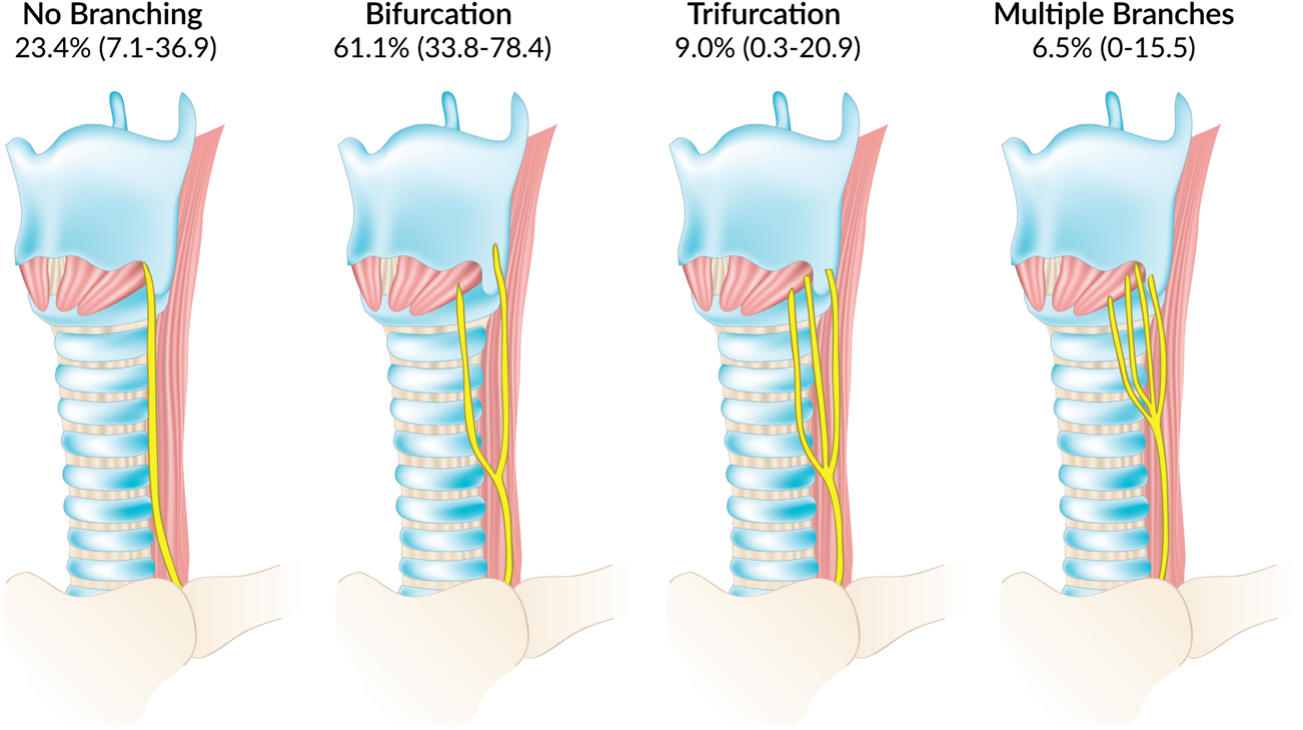
Types of extralaryngeal branching patterns of the recurrent laryngeal nerve with their pooled cadaver prevalence rates. Presented as pooled prevalence rate (95% confidence interval)

A total of 47 studies (*n* = 16,618 nerves) reported data on the type of ELB of the RLN. Bifurcation was the most common pattern observed, with a pooled prevalence of 51.1 % (95 % CI 35.7–55.3) of nerves, followed by no branching, 42.0 % (95 % CI 28.1–47.1) (Online Resource 2). Trifurcation and multiple branches were less common, with pooled prevalence rates of 4.7 % (95 % CI 1.0–9.2) and 2.2 % (95 % CI 0–5.9), respectively. As with the pooled prevalence of ELB, cadaveric and intraoperative studies differed significantly, the rate of bifurcation being significantly greater in cadaveric (61.1 %; 95 % CI 33.8–78.4) (Fig. 3) than intraoperative (37.6 %; 95 % CI 26.2–49.4) studies (Table 4). Detailed subgroup analyses for side, gender, and geographical origin of the study are presented in Table 5. No significant differences were observed in the leave-one-out sensitivity analysis.

**Table 4:** Type of branching by type of study

	No. of studies (no. of nerves)	No branching % (95 % CI)	Bifurcation % (95 % CI)	Trifurcation % (95 % CI)	Multiple branches % (95 % CI)	*I* ^2^: % (95 % CI) *
Overall	47 (16,618)	42.0 (28.1–47.1)	51.1 (35.7–55.3)	4.7 (1.0–9.2)	2.2 (0–5.9)	99.3 (99.2–99.4)
Cadaveric	27 (3361)	23.4 (7.1–36.9)	61.1 (33.8–78.4)	9.0 (0.3–20.9)	6.5 (0–15.5)	99.1 (99.0–99.2)
Intraoperative	19 (13,191)	61.3 (49.1–72.4)	37.6 (26.2–49.4)	1.0 (0–4.1)	0.1 (0–1.7)	99.4 (99.3–99.5)
Intraoperative (prospective)	12 (6644)	65.4 (52.0–77.5)	33.2 (21.2–46.4)	1.2 (0–5.0)	0.1 (0–2.0)	99.0 (98.8–99.2)
Intraoperative (retrospective)	8 (6613)	56.8 (35.5–75.9)	42.1 (22.3–62.4)	0.7 (0–6.2)	0.4 (0–5.1)	99.5 (99.4–99.6)

*p value for Cochran’s *Q* for all subgroups was <0.001

**Table 5:** Type of branching by side, gender, and geographical origin

	No. of studies (no. of nerves)	No branching % (95 % CI)	Bifurcation % (95 % CI)	Trifurcation % (95 % CI)	Multiple branches % (95 % CI)	*I* ^2^: % (95 % CI)*
Overall	47 (16,618)	42.0 (28.1–47.1)	51.1 (35.7–55.3)	4.7 (1.0–9.2)	2.2 (0–5.9)	99.3 (99.2–99.4)
Left sides	26 (3942)	50.7 (33.2–60.6)	38.9 (23.2–49.6)	6.3 (0.7–14.2)	4.1 (0–9.8)	98.5 (98.2–98.7)
Right sides	26 (4262)	45.5 (28.0–56.3)	43.4 (26.2–54.3)	6.8 (0.8–15.4)	4.3 (0–10.5)	98.7 (98.5–98.9)
Males	5 (362)	39.8 (4.5–70.7)	46.8 (8.0–76.2)	9.0 (0–31.8)	4.3 (0–23.0)	97.5 (96.1–98.5)
Females	5 (754)	39.6 (0–76.6)	49.1 (3.6–85.6)	6.2 (0–33.2)	5.1 (0–30.8)	98.4 (97.6–98.9)
Asia	19 (5849)	35.2 (13.1–49.4)	49.9 (23.1–62.2)	8.1 (0–18.6)	6.8 (0–16.6)	99.4 (99.4–99.5)
Europe	16 (5624)	35.7 (18.6–48.4)	59.0 (38.0–69.6)	3.8 (0–10.5)	1.5 (0–6.3)	99.1 (98.9–99.2)
North America	7 (2007)	53.0 (31.3–71.8)	43.4 (22.8–62.9)	2.6 (0–10.9)	1.0 (0–7.1)	98.6 (98.1–99.0)
Oceania	3 (1195)	57.7 (33.5–79.2)	37.0 (15.7–60.3)	5.3 (0–17.9)	0.1 (0–4.4)	97.7 (95.7–98.8)

*p value for Cochran’s *Q* for all subgroups was <0.001

### Distance of extralaryngeal branching site to the inferior rim of the cricothyroid joint

Six studies (*n* = 456 nerves with ELB) reported extractable data for the distance from the ELB site to the inferior rim of the cricothyroid joint. When ELB was present, it occurred most commonly at distance of 1–2 cm, with a pooled prevalence of 74.8 % of cases (95 % CI 44.7–94.1), followed by a distance of 0–1 cm in 15.4 % (95 % CI 0–37.3). Further distance data are presented in Table 6.

**Table 6:** Distance from the extralaryngeal branching site to the inferior rim of the cricothyroid joint

Distance (cm)	Pooled prevalence % (95 % CI)
0–1	15.4 (0–37.3)
1–2	74.8 (44.7–94.1)
2–3	6.0 (0–22.2)
3–4	3.8 (0–17.7)

Six studies (456 nerves with ELB), *I* ^2^ = 97.1 % (95 % CI 95.4–98.1), *p* < 0.001

### Prevalence of positive motor signals in the extralaryngeal anterior and posterior branches

A total of five studies [10, 30, 39, 41, 63] (*n* = 1112 bifurcated nerves) reported the presence of a positive motor signal in the anterior and posterior branches of an extralaryngeally bifurcated RLN, as assessed electrophysiologically during surgical procedures. A positive motor signal was obtained in 99.9 % (95 % CI 99.7–100.0) of anterior RLN branches (*I* ^2^ = 0 % (95 % CI 0–15.4); *p* = 0.912) but in only 1.5 % (95 % CI 0.1–3.9) of posterior branches (*I* ^2^ = 76.6 % (95 % CI 43.0–90.4); *p* = 0.002). Details of the studies reporting on motor signals are presented in Table 7. To mitigate for any potential differences due to recent technical development and changes in electrophysiological equipment, a subgroup analysis restrictive to studies conducted only within the past 2 years was performed. Three studies [30, 39, 41] (*n* = 938 bifurcated nerves) were included in the subgroup analysis. For the anterior branch, a positive motor signal was detected in 99.9 % (95 % CI 99.7–100.0; *I* ^2^ = 0.0 % (95 % CI 0.0–54.3); *p* = 0.454) of cases, equivalent to the overall analysis. For the posterior branch, a positive motor signal was detected in 2.6 % (95 % CI 0.2–6.9; *I* ^2^ = 84.6 % (95 % CI 54.4–94.8); *p* = 0.001) of cases, slightly greater than the overall analysis, albeit not significantly.

**Table 7:** Motor signaling in extralaryngeal branches of the recurrent laryngeal nerve

Study ID	Method of signal detection	*n* (number of RLN with ELB)	Positive motor signal in anterior branch (%)	Positive motor signal in posterior branch (%)
Barczyński et al. [30]	NIM 2.0 followed by the NIM 3.0 system (Medtronic USA, Inc., Jacksonville, FL) at 1 mA	613	613 (100 %)	8 (1.3 %)
Gurleyik [41]	IONM device. Nerve Integrity Monitor (NIM-Response 3.0 System; Medtronic Xomed, Jacksonville, FL) at 1 mA	61	61 (100 %)	7 (11.5 %)
Fontenot et al. [39]	IONM device (Xomed NIM System; Medtronic USA, Inc., Jacksonville, FL) at 1.0 mA	264	264 (100 %)	3 (1.1 %)
Kandil et al. [10]	IONM device (Xomed NIM System; Medtronic USA, Inc., Jacksonville, FL) at 0.5 mA	133	133 (100 %)	0 (0 %)
Serpell et al. [63]	IONM device (Xomed NIM System; Medtronic USA, Inc., Jacksonville, FL)	41	41 (100 %)	0 (0 %)

## Discussion

There is wide variability in the ELB of the RLN, and its characteristics have not been assessed completely. The aim of our study was to provide a comprehensive meta-analysis on the ELB variants of the RLN to allow for pertinent clinical applications of the data.

Our results showed that the overall prevalence of ELB was 60.0 %. Studies such as Dai et al. [38] and Cakir et al. [33] demonstrated similar findings, whereas other studies have reported prevalences ranging anywhere from 5 % [11] to 100 % [12–21]. Extensive subgroup analysis on the presence of ELB was performed. There were significant differences in the prevalence of ELB between cadaveric studies (73.3 %) and intraoperative studies (39.2 %). This suggests that the prevalence of ELB could be grossly underestimated in the operating theater. We believe this could be due to difficulty in viewing the branches of the RLN because of localized inflammation, edema, and the small caliber of nerves exhibiting ELB. Furthermore, the inability to completely dissect the small branches during operations and surgeons not addressing the small “accessory” RLN branches as ELB may also contribute to the large gap between cadaveric vs. intraoperative prevalence. Some intraoperative studies noted that only nerves which bifurcated and entered at the lower margin of the larynx were indeed counted as branched nerves (ELB) [30, 39]. An intraoperative study by Gurleyik in 2013 [40] noted that many of the small branches of the RLN that may be found in cadaveric studies are simply not perceivable during surgical procedures. As is mentioned in a study by Ngo Nyeki in 2015 [54], intraoperative assessment of ELB was not systematically investigated. Regardless, diligent assessment and dissection are needed during surgical procedures to avoid iatrogenic injuries and complications. Future research regarding RLN ELB needs to be meticulous, particularly with regard to intraoperative studies. We believe that, to date, the true prevalence has been better reflected in the results of cadaveric-based studies. Furthermore, detailed morphometric analysis could be performed on the RLN to determine which, if any, caliber nerve is likely to have ELB.

No major deviations from the overall prevalence were noted in geographic, sex-based, and laterality subgroup analyses, and thus, all patients should be considered to have equal risk of ELB. We posit that this is a logical finding, given that the embryological development of these structures is largely uniform, barring any unique developmental pathologies. Importantly, surgeons need to refrain from assuming that the presence of ELB is always purely symmetrical. We note that a mere 36.5 % of RLNs had symmetrical branching.

A subset of the studies [4–7, 9–21, 26–30, 32–36, 38–43, 47, 48, 50–52, 54, 55, 59, 60, 62, 63, 66, 68, 71, 73, 75–82] reporting information on ELB also reported information on the type of branching. In those studies, bifurcation was the most common pattern observed, with a pooled prevalence of 51.1 % of nerves. The second most common pattern was the lack of branching, followed by trifurcation and multiple branches. We note that due to the potential lack of systematic investigation of ELB during intraoperative procedures, there may be underreporting of non-bifurcating patterns (i.e., trifurcation, multiple branches) and ELB in general. As was mentioned previously, some surgeons only considered ELB to be present in cases of bifurcation as is seen in Barczyński et al. [30] and Fontenot et al. [39], and thus, some patterns of trifurcation and multiple branching may have been overlooked or not have been noticeable.

A small number of studies reported on the distance of the branching point of the RLN from the inferior rim of the cricothyroid joint (CTJ). Most of the nerves (90.2 %) branched within the proximal 2 cm of the CTJ, suggesting that some intraoperative studies have failed to note incidences of branching if it occurred in such close proximity to the larynx. With the overwhelming number of nerves branching at this distance from the cricothyroid joint, it is evident how essential it is to successfully identify the RLN in the surgical field.

Motor signaling was assessed in both anterior and posterior divisions of the RLN. It was noted that nearly 100 % of anterior branches had positive motor signaling, but it was found posteriorly in only 1.5 %. This supports the notion that the anterior branch is almost the sole supplier of motor innervation to the RLN-innervated muscles of the larynx, the sensory fibers traveling in the posterior division [10]. However, our analysis notes that up to 3.9 % of posterior branches could contain some motor fibers and, as such, care should be taken to protect them whenever possible. Additional problems arise when looking at the rapid development and honing of the technology and devices used to identify these signals. The more recent studies included in our analysis [30, 41] tended to have higher incidence of motor signaling in the posterior branches leading to the conclusion that signals in this division may have been previously underreported due to devices simply not being sensitive enough.

Injury to the RLN is one of the most feared and challenging postoperative complications in thyroid surgery, with 6 % experiencing temporary deficits and 1 % experiencing permanent nerve palsy [76, 77]. With the realization that over half of patients have some element of ELB, precautions should be taken to prevent these iatrogenic complications directly. We recommend that surgeons attempt to expose the RLN completely, along with any of its early bifurcating terminal branches if they are present [41]. However, not all surgeons recommend complete dissection of the RLN, as it may require a more invasive procedure [54].

The use of anatomical landmarks such as the nerve’s relationship with the inferior thyroid artery, ligament of Berry, tracheoesophageal groove, or tubercle of Zuckerkandl may be helpful for determining the RLN’s location but are often highly variable in their anatomical relationship to the nerve [40, 54, 78]. These relationships may be further complicated by present pathology such as a large goiter or inflammation and edema altering the normal anatomy, making nerve identification more difficult. The use of intraoperative nerve monitoring (IONM) devices has shown potential; however, to date, it is not significantly more effective at reducing iatrogenic injuries as compared to nerve visualization [79, 80]. We make the proposition that IONM be used in instances where patients may have an underlying pathology such as large goiter or inflammation which restricts visualization of the necessary structures or, when patients are undergoing reoperation, as scar tissue can make nerve identification difficult [81]. As is noted in the German Association of Endocrine Surgeons’ guidelines for thyroid disease, the use of IONM should serve as a complimentary tool for surgeons for ensuring the identification and protection of the RLN during operative procedures [83]. Further described by Musholt et al. [83] is that IONM is indispensable in the prevention of bilateral RLN injuries which result in severe deficits. Another option available to surgeons is the use of pre-operative ultrasonography (USG) to identify structures and potential anatomical variants. Rare variants such as nonrecurrent laryngeal nerves have been successfully identified using USG 98 % of the time, and thus, this method may be helpful in reducing the risk of iatrogenic injury to the RLN [82]. Konschake et al. [84] and Gong et al. [85] note the use of USG as the most efficient and effective way of preoperatively identifying these variants and avoiding unnecessary radiation exposure. The use of pre-operative USG to identify ELB should be evaluated in future studies.

Our meta-analysis on the ELB of the RLN was limited by a number of factors, such as unclear or difficult-to-interpret data and the lack of detailed information on nerve branching patterns, which resulted in the exclusion of several studies. Additionally, there was high heterogeneity among studies, which persisted despite extensive subgroup analysis, suggesting it could be attributed to the intrinsic variability of the RLN. Further limitation factors included the lack of a quality assessment and risk of bias tool for anatomical studies and a lack of assessment of publication bias because there was no statistical measure for prevalence meta-analysis. Throughout the study, authors were contacted when necessary and possible in an attempt to resolve discrepancies, provide clarification, and minimize bias.

In conclusion, the RLN is highly variable and has a high prevalence of ELB. The RLN in most of the population has ELB in the form of bifurcation, followed in prevalence by no branching, trifurcation, and multiple branching. Extralaryngeal branching, if present, is typically within 2 cm of the inferior rim of the cricothyroid joint, with the overwhelming majority of anterior branches containing the motor fibers and posterior branches the sensory fibers. The high prevalence of ELB needs to be factored into the assessment and operative procedure of every patient. Only a proper and complete understanding of the variant anatomy of the RLN can provide for the best chance of a complication- and injury-free procedure.

## Electronic supplementary material

Below is the link to the electronic supplementary material.

Online Resource 1 PRISMA 2009 Checklist (PDF 75 kb)

Online Resource 2 Forest Plots for Types of Branching (PDF 178 kb)
