# Supplementary material for: Extralaryngeal branching of the recurrent laryngeal nerve: a meta-analysis of 28,387 nerves
Source: Langenbecks Arch Surg. 2016 Jun 2;401(7):913–23. doi: 10.1007/s00423-016-1455-7 (PMC5086344; doi:10.1007/s00423-016-1455-7)

Online Resource 3 - Forest Plots for Types of Branching

No Branching

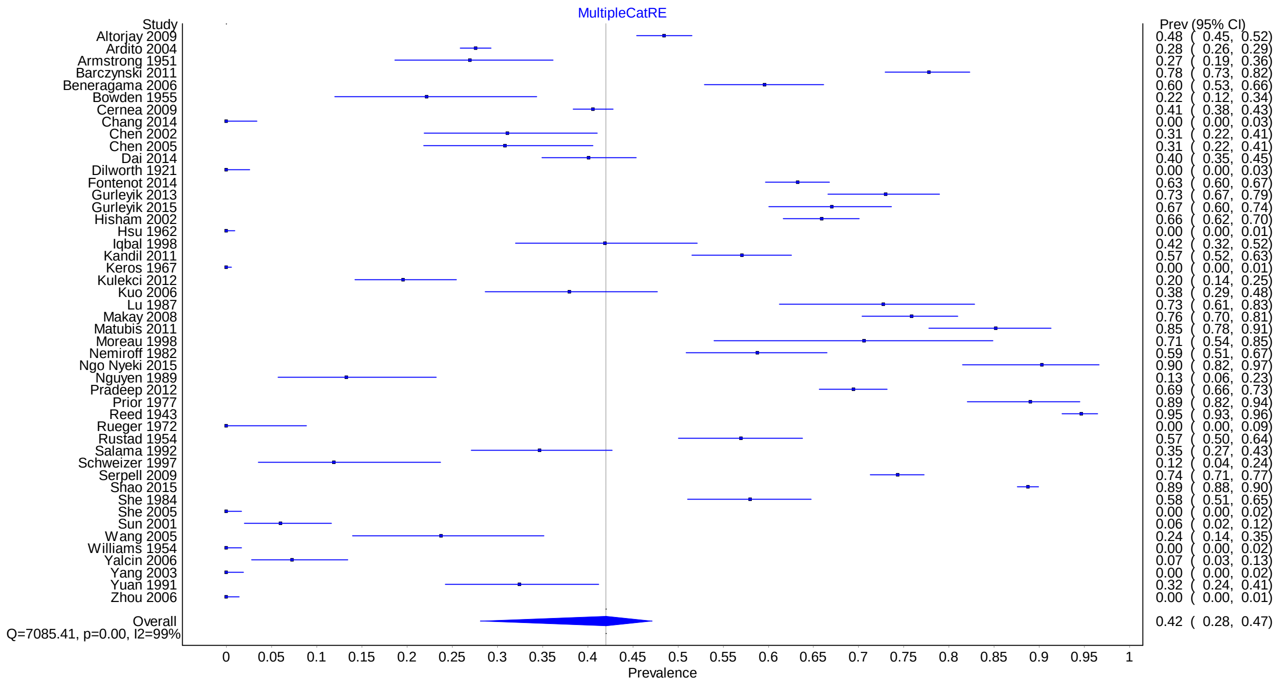

Bifurcation

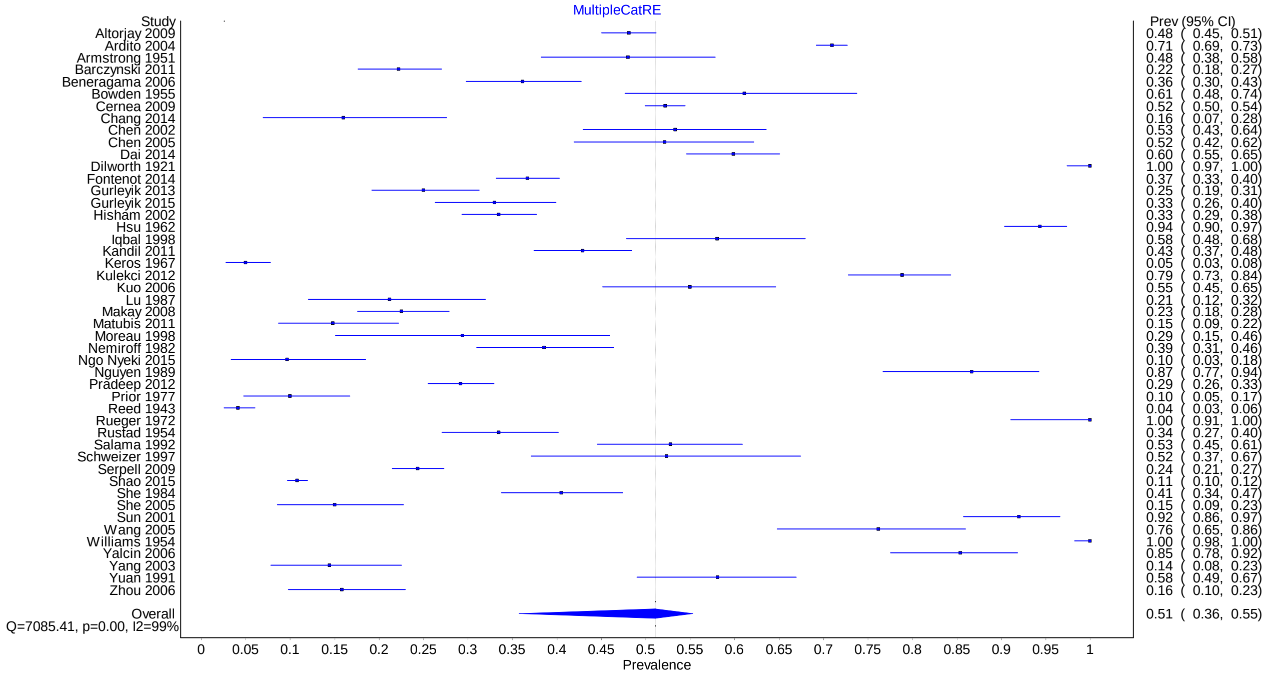

Triplication

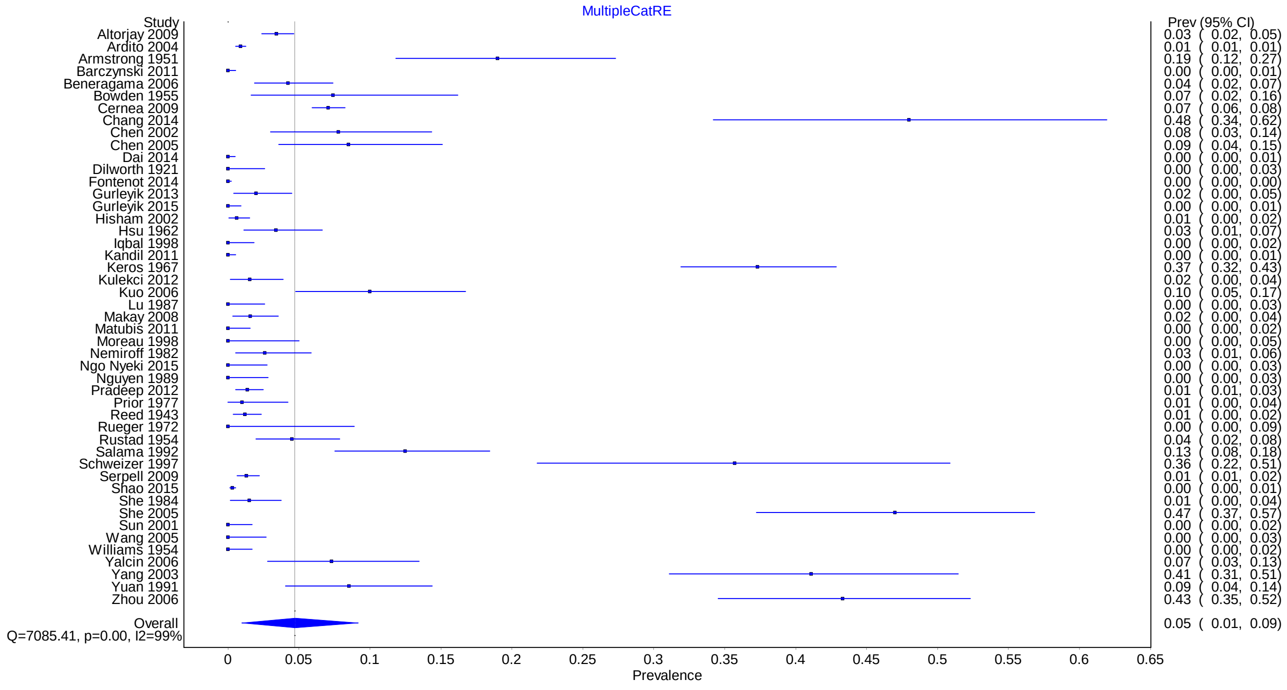

Multiple Branches

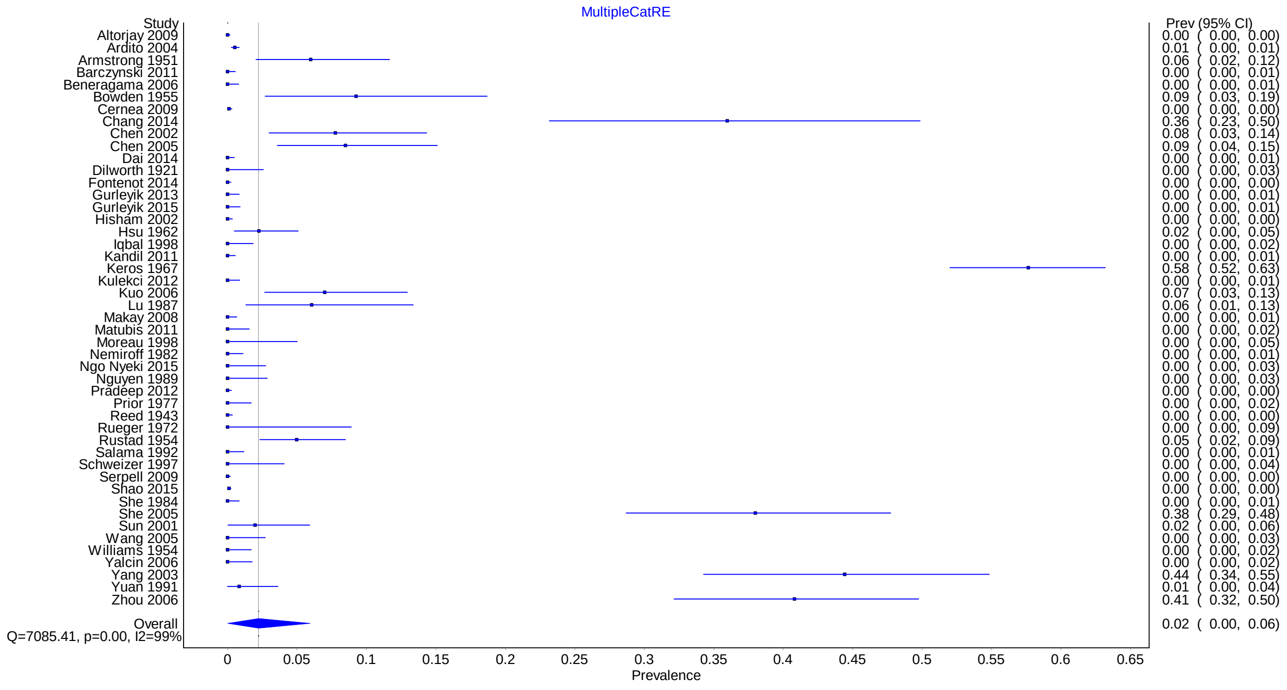

Supplement: Supplementary file 2 — Forest Plots for Types of Branching (PDF 178 kb) [file 423_2016_1455_MOESM2_ESM.pdf]
